# Enhancing osteoblast survival through pulsed electrical stimulation and implications for osseointegration

**DOI:** 10.1038/s41598-021-01901-3

**Published:** 2021-11-17

**Authors:** Emily Pettersen, Furqan A. Shah, Max Ortiz-Catalan

**Affiliations:** 1 Center for Bionics and Pain Research, Mölndal, Sweden; 2 Department of Electrical Engineering, Chalmers University of Technology, Gothenburg, Sweden; 3 Center for Advanced Reconstruction of Extremities (C.A.R.E.), Sahlgrenska University Hospital, Mölndal, Sweden; 4 Department of Biomaterials, Institute of Clinical Sciences, Sahlgrenska Academy, University of Gothenburg, Gothenburg, Sweden; 5 Department of Orthopaedics, Institute of Clinical Sciences, Sahlgrenska Academy, University of Gothenburg, Gothenburg, Sweden

**Keywords:** Implants, Biomedical engineering

## Abstract

Electrical stimulation has been suggested as a means for promoting the direct structural and functional bonding of bone tissue to an artificial implant, known as osseointegration. Previous work has investigated the impact of electrical stimulation in different models, both in vitro and in vivo, using various electrode configurations for inducing an electric field with a wide range of stimulation parameters. However, there is no consensus on optimal electrode configuration nor stimulation parameters. Here, we investigated a novel approach of delivering electrical stimulation to a titanium implant using parameters clinically tested in a different application, namely peripheral nerve stimulation. We propose an in vitro model comprising of Ti6Al4V implants precultured with MC3T3-E1 preosteoblasts, stimulated for 72 h at two different pulse amplitudes (10 µA and 20 µA) and at two different frequencies (50 Hz and 100 Hz). We found that asymmetric charge-balanced pulsed electrical stimulation improved cell survival and collagen production in a dose-dependent manner. Our findings suggest that pulsed electrical stimulation with characteristics similar to peripheral nerve stimulation has the potential to improve cell survival and may provide a promising approach to improve peri-implant bone healing, particularly to neuromusculoskeletal interfaces in which implanted electrodes are readily available.

## Introduction

Osseointegration^1^ is the direct structural and functional bonding between an implant surface and bone, and has had a substantial effect on dental and orthopaedic rehabilitation. In comparison to fitting a limb socket prosthesis over soft-tissue, osseointegration allows for skeletal fixation, resulting in a more comfortable and effective mechanical coupling to transfer load between an artificial limb and the human body^2^. The artificial limb is connected to the bone via an implant system with implanted and percutaneous components, known as *fixture* and *abutment*, respectively. The fixture is the component that osseointegrates within the bone intramedullary canal. The abutment extends from inside the fixture and through the skin to provide mechanical connection for the prosthesis. Recently, one such implant system has been developed that also includes neuromuscular electrodes to record bioelectric signals for control of the artificial limb, and to deliver electrical stimulation to severed nerves for eliciting sensory feedback^3^.

Commercially-pure titanium and titanium alloys (typically Ti6Al4V) are most frequently used in load-bearing orthopaedic implants due to their biocompatibility, mechanical strength, high corrosion resistance and unique ability to osseointegrate^4,5^. After surgical implantation of an orthopaedic fixture, there is typically a healing period of 3–12 months prior to loading^2,6,7^. During which time bone adaptation (osseointegration) to the implant surface occurs; an ideal fixation would mitigate any movement at the bone-implant interface^1^. Various factors affect peri-implant healing, including implant design and host bone quality^8^. In conditions where early implant loading is desired, or when the implant is placed in compromised healing conditions, there is a need to stimulate the progression of osseointegration^9,10^. Reduced healing time, early restoration of function, and prolonged effective lifespan of the prosthesis are the main driving forces behind enhancing osseointegration at the bone-implant interface. To this end, various approaches have been undertaken, including development of different metal alloys, use of macro-porous geometries, manufacturing techniques, surgical techniques, and alteration of implant surface properties such as topography and chemistry^11^.

Clinically, electrical stimulation has been instrumental in the treatment of a wide spectrum of disorders and disabilities^12^. Implantable devices that deliver electrical stimulation have shown successful outcomes in applications such as cochlear implants to restore hearing function^13^, wound-healing therapies intended for the closure of chronic wounds^14^, and in limb prostheses to restore sensory perception^3,15^. Electrical stimulation to promote osteogenesis for bone fracture healing has been recognised since the 1950s^16^, and explored for bone injury treatments including bone healing of union and non-union fractures^17^. Furthermore, electrical stimulation has been investigated as a potential treatment for bone ingrowth into implants, in vitro and in vivo*.* Different approaches have been developed by varying the electrode configuration, current type and source, and electrical stimulation parameters (e.g., amplitude and frequency)^18,19^. Three modalities of electrical stimulation have commonly been used for this purpose: (i) direct stimulation, (ii) indirect stimulation (capacitive or inductive couplings), and (iii) combined stimulation^20^. Studies reveal significant increases in bone-implant contact^4,9,21–23^, differentiation of preosteoblasts^12,24^, and increased cell proliferation^25^ upon application of direct current (DC) stimulation. However, DC stimulation can include pH shifts, accumulation of oppositely charged proteins at the implant surface, and production of reactive oxygen species in the adjacent environment^25^. Pulsed electrical stimulation overcomes some of these challenges^9,25^, particularly where pulses of opposite magnitude are used to balance the displacement of charges^26^. Pulsed electrical stimulation has shown beneficial effects on cell proliferation, in vitro^25^ and bone-implant contact, in vivo^9^. However, further investigation of the optimal electrical stimulation parameters is needed^18,19^.

In this work, we investigated the response of MC3T3-E1 preosteoblasts (precursor cells to osteoblasts) to pulsed electrical stimulation with parameters similar to those used in artificial limbs for sensory feedback through peripheral nerve stimulation^3,15,26^. We utilised parameters that have been used safely with implanted electrodes for several years^3^ and are compatible with electronic embedded system for artificial limbs^27^. A versatile in vitro setup comprising a bespoke, 3D-printed poly(lactic acid) (PLA) chamber was developed to minimise risk of inadvertent motion and enable reproducible positioning of individual components. Flat Ti6Al4V plates represent the implant to be osseointegrated and Ti6Al4V discs represent implanted electrodes that serve as the electrical reference. MC3T3-E1 preosteoblasts cultured on the flat Ti6Al4V plates were exposed to different combinations of pulse amplitude and frequency over a continuous 72-h period. Thereafter we evaluated the pH, cell survival, and collagen production and compared to unstimulated controls (Ctrl). Our results show, for the first time, that pulsed electrical stimulation significantly accelerates collagen production of MC3T3-E1 cells, which is contingent on osteogenic differentiation. In addition, electrical stimulation significantly improves cell survival, without detectable changes in the local pH.

## Results

In vitro pulsed electrical stimulation experiments were conducted using a PLA chamber with integrated features for positioning the implants with precultured cells and reference electrodes. MC3T3-E1 cells were precultured on Ti6Al4V implants for 16 h prior to 72 h of culture exposure with and without electrical stimulation. Three combinations of pulse amplitude (10 µA or 20 µA) and frequency (50 Hz or 100 Hz) were tested; A10F50 (amplitude 10 µA, frequency 50 Hz), A20F50 (amplitude 20 µA, frequency 50 Hz), and A20F100 (amplitude 20 µA, frequency 100 Hz). Compared to Ctrl, no significant changes in pH were detected for A10F50 (*p* = 0.931), A20F50 (*p* = 0.259), or A20F100 (*p* = 0.847) after 72 h of electrical stimulation (16 h of preculture + 72 h of stimulation).

### No morphological changes after 72 h of pulsed electrical stimulation

**Figure 1:**
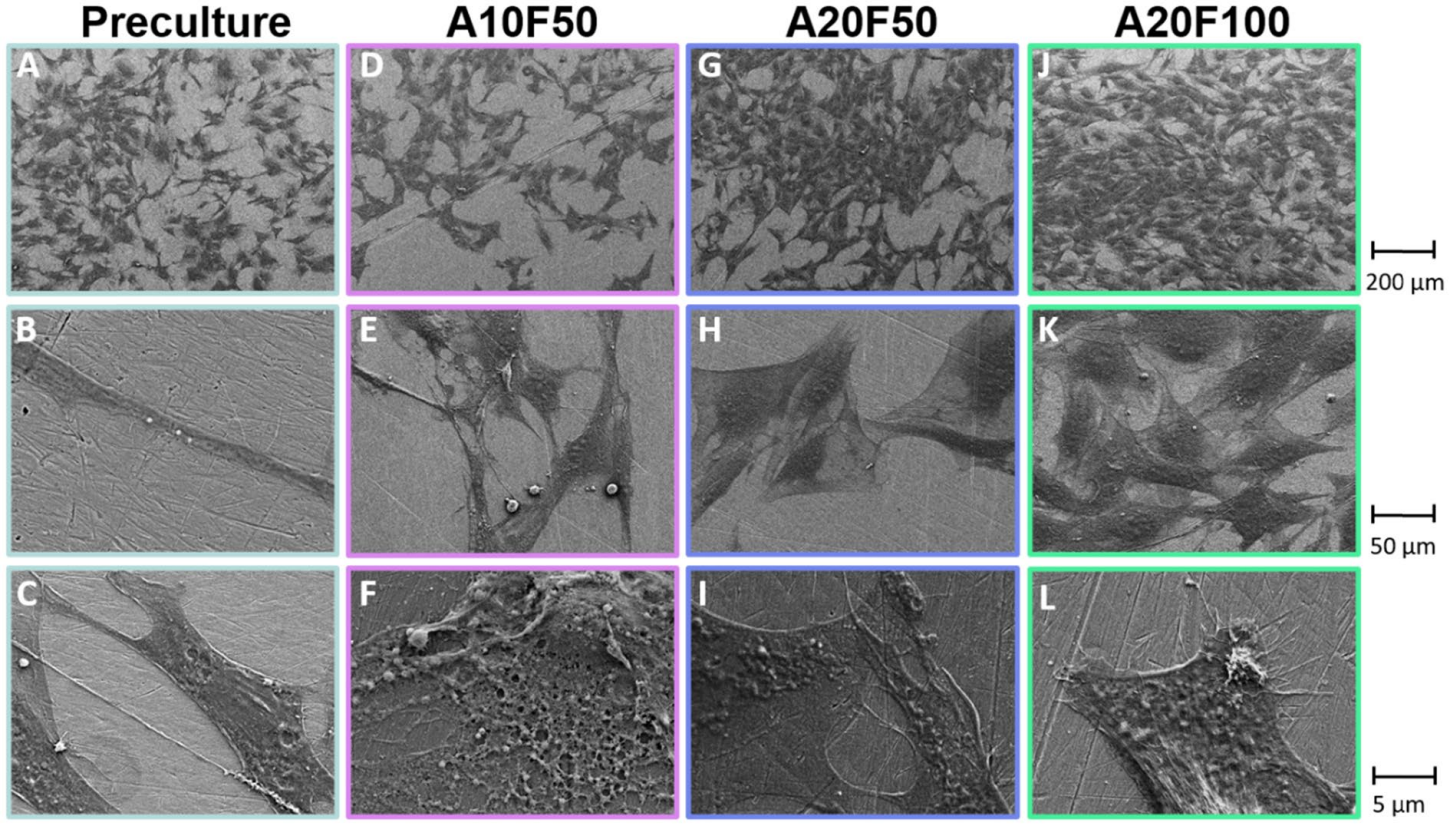
MC3T3-E1 preosteoblast morphology (scanning electron microscopy). (**A**–**C**) MC3T3-E1 cells precultured cells on Ti6Al4V for 16 h prior to electrical stimulation. (**D**–**L**) MC3T3-E1 cells after 72 h of pulsed electrical stimulation under different combinations of amplitude and frequency; (**D**–**F**) A10F50, (**G**–**I**) A20F50, and (**J**–**L**) A20F100.

In the preculture group—the group cultured for 16 h prior to stimulation, the MC3T3-E1 cells were elongated in appearance (Fig. 1A–C). After 72 h of pulsed electrical stimulation, the MC3T3-E1 cells displayed a flattened morphology (Fig. 1E,H,K) and the cell density was higher in A20F50 and A20F100 compared to A10F50 (Fig. 1D,G,J). The stimulated groups (A10F50, A20F50, and A20F100) displayed extracellular matrix-like features (Fig. 1F,I,L), of which the highest density was observed for the A20F50, using scanning electron microscopy (SEM).

### Pulsed electrical stimulation improves cell survival and collagen production

**Figure 2:**
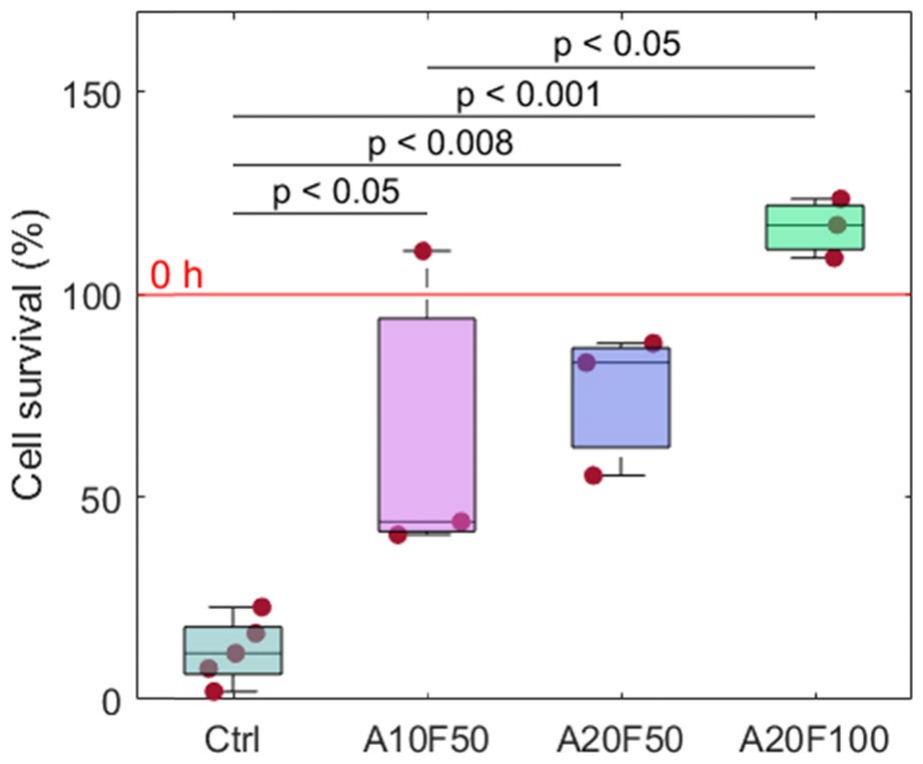
MC3T3-E1 cell survival at 72 h. The average number of implant-adherent cells at 16 h preculture (~ 24,600 cells per implant) is taken as 100% survival at 0 h. Ctrl, n = 5; A10F50, A20F50, and A20F100, n = 3.

Compared to the Ctrl (Fig. 2), we found significantly improved cell survival in the stimulated groups A10F50 (*p* < 0.05), A20F50 (*p* < 0.008) and A20F100 (*p* < 0.001). No significant difference in cell viability was observed between the groups stimulated with 50 Hz frequency (i.e., A10F50 vs. A20F50). Furthermore, no significant difference in cell viability was observed between the groups stimulated with 20 µA amplitude (*p* = 0.121) (i.e., A20F50 vs. A20F100). Moreover, there was a significant difference between A10F50 and A20F100 (*p* < 0.05*).* Specifically, the cell population recorded for A20F100 exceeded the number of cells at 0 h.

**Figure 3:**
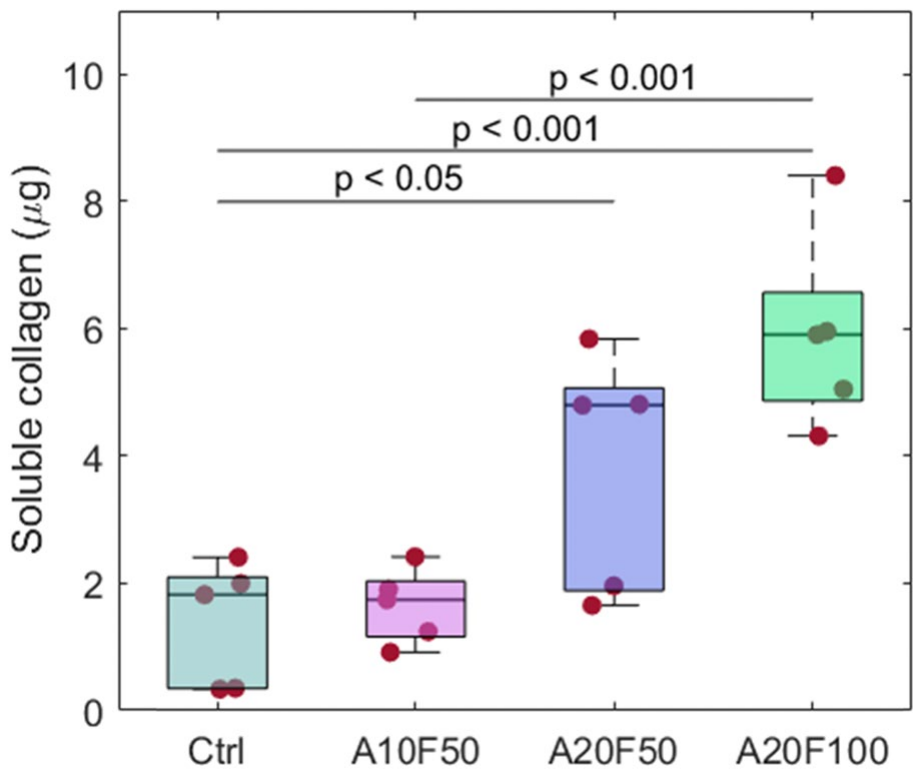
Collagen production at 72 h, Ctrl, A10F50, A20F50, and A20F100, n = 5.

Compared to Ctrl, collagen production was significantly higher for A20F50 (*p* = 0.0364) and A20F100 (*p* < 0.001), but not for A10F50 (*p* > 0.05) (Fig. 3). The amount of collagen detected was highest for A20F100, and a significant increase was observed between A10F50 and A20F100 (*p* < 0.001), however, no significant difference was observed between groups stimulated at 50 Hz frequency (*p* = 0.0873) (i.e., A10F50 vs. A20F50) or between groups stimulated at 20 µA amplitude (*p* = 0.0979) (i.e., A20F50 vs. A20F100).

**Figure 4:**
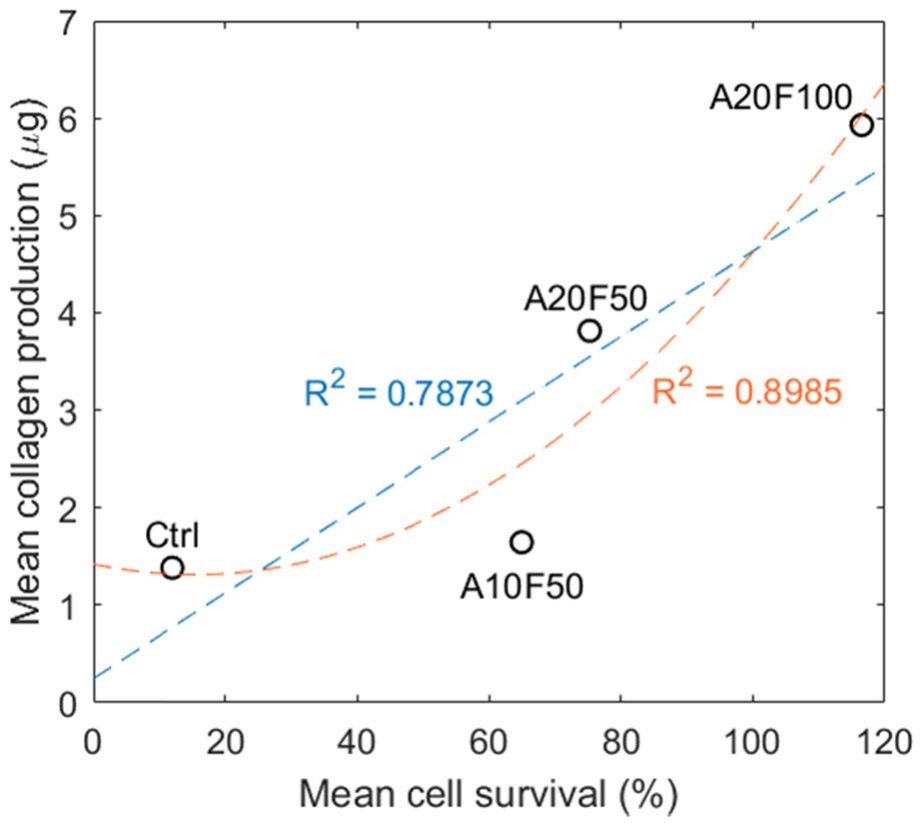
Linear regression plot and polynomial plot. Mean collagen production (µg) in each group is plotted against mean cell survival (%) in each group. Linear fit: y = 0.00018 + 0.25; Polynomial fit: y = 8 × 10^−9^x^2^ – 6 × 10^−5^x + 1.4232.

Interestingly, a nonlinear relationship was noted between cell survival and collagen production (Fig. 4), which warrants further investigation.

## Discussion

Electrical stimulation has been regarded as a potential approach for promoting peri-implant osteogenesis^18,19^. Here, we investigated the impact of pulsed electrical stimulation of similar characteristics as used in artificial limbs to restore sensory feedback through peripheral nerve stimulation^3,15,28^ on MC3T3-E1 preosteoblasts. We employed an in vitro setup comprising Ti6Al4V implants that were precultured with MC3T3-E1 cells, stimulated for 72 h at two different pulse amplitudes at two different frequencies. We demonstrated that pulsed electrical stimulation enhances cell survival and collagen production compared to unstimulated controls in a dose-dependent manner.

Across the implant surface, osteoblasts displayed a characteristic flattened and stretched appearance. There were no dissimilarities in cell morphology between the various stimulated groups, indicating that the different electrical stimulation combinations did not have a visual impact on the cell morphology compared to each other. Some extracellular matrix-like features were noted on the implant surface under electrical stimulation. However, further investigation is required to better understand the structural and functional characteristics of extracellular matrix produced under pulsed electrical stimulation.

We used flat Ti6Al4V plates to mimic the implant for two reasons: (1) The model was limited to a 2D culture in a homogenous environment; (2) It was not known how far from the implant surface the cell responsiveness to pulsed electrical stimulation would be. However, this work can be developed to replicate (cylindrical) upper limb prosthesis fixtures (40 mm long and 4 mm in diameter) with minor modifications to the experimental setup^29^.

Cell counts at 72 h for Ctrl, A10F50 and A20F50 were lower than at 0 h (i.e., start of stimulation). This may be explained by the use of HEPES buffer, for extended periods outside of CO_2_ incubators, which could have caused non-physiological fluctuations in pH^30^. In addition, other contributing factors to reduced cell survival could have been poor cell attachment to the implant after 16 h of preculture and physical cell removal during transfer from preculturing tube to chamber prior to stimulation start. However, the decrease in cell number was significantly less in the stimulated groups A10F50, A20F50 and A20F100 compared to control, meaning significantly higher cell survival in stimulated groups. The mean value in A20F50 was larger compared to A10F50, although there was no significant difference between the amplitude values. Comparing the two frequencies, 50 and 100 Hz, there was no significant increase in cell survival in A20F100 compared to A20F50, although A20F100 was the only group where the cell population had increased from that at time point 0 h. Applying higher frequency of the pulse train means that the time period between each pulse event decreases, and thus the stimulation begins to resemble DC.

The groups stimulated with 20 µA amplitude, A20F50 and A20F100, showed significantly accelerated collagen production compared to Ctrl. Furthermore, A20F100 showed a significant increase in collagen production compared to A10F50. Soluble collagen production is produced by osteoblasts^31^ and the cell line used here, MC3T3-E1, is preosteoblastic. Therefore, the current findings also suggest that electrical stimulation has a positive influence on osteogenic differentiation of preosteoblasts. Interesting future directions would be to investigate whether the electrical stimulation itself promotes differentiation in basal (or non-osteogenic) media, and also if electrical stimulation drives differentiation of mesenchymal stem cells to osteoblasts. Additional future directions include model development with cylindrical implant fixtures, as well as to investigate parameters such as pulse width, duty cycle, and stimulation duration longer that 72 h. Furthermore, gene and protein expression of relevant bone markers, as well as investigation of underlying mechanisms including signalling pathways of bone morphogenetic proteins (BMPs) and Wingless and Int (WNT) would be of interest.

## Conclusions

In summary, pulsed electrical stimulation exhibited a strong positive influence on osteoblast survival (and/or attachment) on Ti6Al4V surfaces and collagen production, which are important processes in osseointegration. Our results showed enhanced cell survival with stimulation of 10 µA and 20 μA and bone cells grew in higher numbers on stimulated Ti6Al4V compared to unstimulated Ti6Al4V. Among all test conditions, 20 μA indicated a beneficial amplitude value, particularly at a frequency of 100 Hz. More specifically, 100 Hz frequency was found to favour cell proliferation in comparison to not only unstimulated conditions but also stimulation at 50 Hz. In addition to the highest osteoblast density, stimulation at 20 μA and 100 Hz also led to five times more collagen production at 72 h compared to unstimulated conditions.

Therefore, it can be concluded that pulsed electrical stimulation with characteristics similar to sensory feedback stimulation in artificial limbs, has a beneficial impact on cell survival and collagen production. These preliminary findings offer insight into a promising novel approach towards improving peri-implant bone healing, i.e., osseointegration. Important applications would be stimulation to reduce healing time and restore early function or regain osseointegration of failing bone-anchored implants.

## Materials and methods

### Experimental setup

The in vitro experimental setup included a 3D-printed chamber made of poly(lactic acid) (PLA), a plate and two discs made of Ti6Al4V, a bipolar constant current stimulator (DS5, Digitimer Ltd., UK) and an Arbitrary Function Generator (AFG-2112—12 MHz, GW Instek, Taiwan).

**Figure 5:**
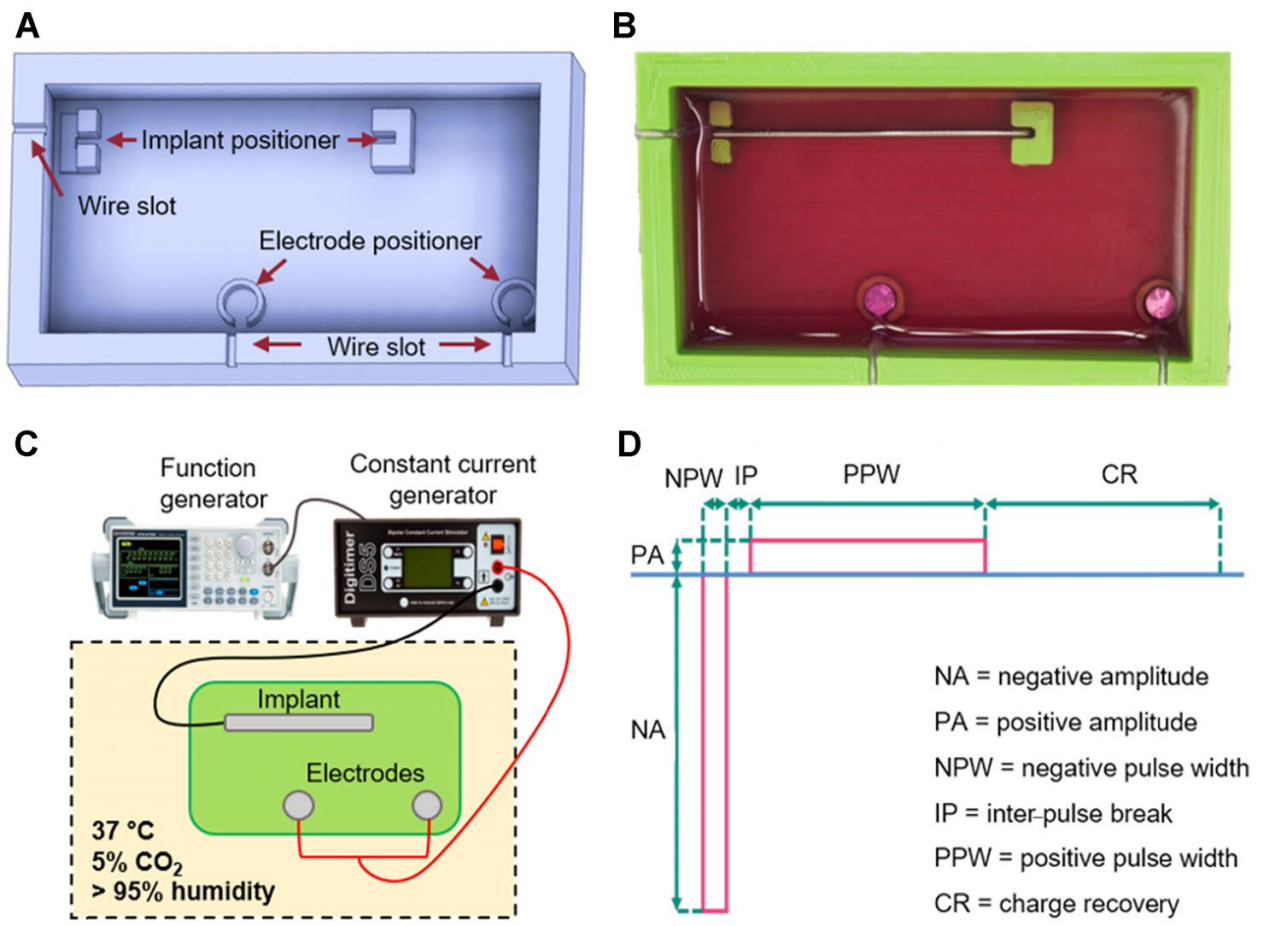
Experimental setup. (**A**) CAD sketch of the 3D-printed chamber where specific design features are indicated by arrows. (**B**) Photograph of the experimental setup during the experiments where the implant and electrodes are placed in their positioners and the chamber is filled with cell culture medium. (**C**) The experimental electrical circuit where the implant is the cathode while the electrodes function as anodes connected to the constant current generator, which in turn is connected to the function generator. (**D**) Pulse characteristics.

The PLA chamber was a rectangular box with integrated design features (Fig. 5A,B). The chamber contained two different types of positioners, one for the implant and two for the electrodes. The implant positioner had two components that allowed the implant to stand up by sliding into two slots. The slot nearest to the chamber wall was designed with an outlet that allowed the wire to exit the chamber. The electrode positioner contained a cylindrical extrusion with an opening closest to the wall in order to allow the wire to exit the chamber. Three rectangular slots were integrated into the upper surface of the chamber wall, two at the long side and one at the short side. Those slots were designed to prevent rotation and restrict movement of the implant and/or the electrodes during the experiment.

The Ti6Al4V plate (40 mm long, 4 mm wide, and 1 mm thick) was chosen to imitate the implant fixture (since they are the same length and the diameter is the same as the flat plate width). Ti6Al4V discs (4 mm diameter and 3 mm high) were chosen to act as electrodes. The wires (10 mm long) connecting the Ti6Al4V plate and discs to the current generator were made of titanium grade 1 (Sargenta AB, Malmö, Sweden). The wire segment directly exposed to the cell culture medium was isolated within silicone tubing. To prevent corrosion between the wire and the Ti6Al4V plate and wicking of cell culture medium into the silicone tubing, a small droplet of silicone adhesive (Med-1037, NuSil^®^, Avantor^®^) was used to cover the weld and to seal the silicone tubing. The function generator was used to control the bipolar constant current generator that sent out pulses of desired characteristics. The Ti6Al4V plate was connected to the negative output, thus functioning as a cathode, and the electrodes were connected to the positive output, thereby serving as anodes (Fig. 5C).

### Expansion of MC3T3-E1 cells and preculture on Ti6Al4V

The same vial of passage 10 osteoblastic cell line MC3T3-E1 subclone 14, established from C57BL/6 mouse (*Mus musculus*) calvaria, obtained from ATCC^®^, was used for every experimental cycle. Cells were precultured on the Ti6Al4V plate surface in a 2 mL polypropylene, screw cap micro tube (Sarstedt AG & Co. KG, Germany) in a lying position at 37 °C in 95% humidity and 5% CO_2_ for 16 h with Dulbecco’s Modified Eagle’s Medium (DMEM, Gibco™, USA) containing 4.5 g/L d-glucose, l-glutamine, and 25 mM 4-(2-hydroxyethyl)-1-piperazineethanesulfonic acid (HEPES) buffer and supplemented with 10% foetal bovine serum, 1% penicillin–streptomycin, and 0.25 mg/mL amphotericin-B (sDMEM). The Ti6Al4V plate surface facing the electrodes in the experimental setup was placed upwards at a cell seeding density of 10^5^ cells per implant. Six randomly-selected, precultured Ti6Al4V plates were counted at 16 h to determine number of cells attached to the surface prior to start of stimulation and two randomly-selected Ti6Al4V plates were qualitatively investigated using SEM after 16 h of preculturing before stimulation.

The Ti6Al4V plate with precultured cells was removed from the micro tube and carefully positioned in the PLA chamber. The electrodes were placed in their positioners and connected to the generators. 12 mL osteogenic differentiation media (sDMEM supplemented with 1% l-ascorbic acid 4.5 mM, 1% dexamethasone 1 mM and 2% β-glycerophosphate 1 M) were added to the chamber before placement in a non-CO_2_ incubator (Heratherm IMC 18, Thermo Scientific). The experiment started when electrical stimulation was applied.

### Pulsed electrical stimulation

The electrical stimulation consisted of charge-balanced, cathodic, rectangular, biphasic asymmetric (10:1), current-controlled pulses (Fig. 5D). The cathodic phase (negative pulse) was followed by an inter-pulse break (zero amplitude) and a recovery phase (positive pulse) that was 10 × smaller in amplitude and 10 × longer in duration than the cathodic phase. Each stimulation pulse was followed by a charge recovery phase where any residual charge was recovered back to zero to ensure that charge accumulation cannot occur.

### Stimulation treatment

Pulsed electrical stimulation was applied for a continuous duration of 72 h at three combinations of negative pulse amplitude (denoted “A”, 10 and 20 μA) and frequency (denoted “F”, 50 and 100 Hz), e.g., A10F50, A20F50, A20F100. Fixed pulse parameters included negative pulse width (500 μs), inter-pulse break (50 μs) and sample frequency (100 kSPS). To adjust for evaporation, 2 mL of fresh medium were added per chamber every 24 h. The three first replicates in each stimulated group were evaluated for cell count and the two last replicates was prepared for SEM imaging. Every replicate was evaluated for collagen production.

## Evaluation assays

### Cell distribution, morphology, and attachment

Distribution, morphology, and attachment of cells on the titanium implant were qualitatively evaluated using SEM imaging (*n* = 2 per group). Samples were fixed in 4% paraformaldehyde for two hours at room temperature and stained with 1% OsO_4_ for two hours. After rinsing with 0.15 M Na-cacodylate buffer, the samples were briefly dehydrated in a graded ethanol series for five min at each step (50, 70, 80, 90, 95 and 100% ethanol) and allowed to air dry. The samples were sputter-coated with gold before examination in an Ultra 55 FEG SEM (Leo Electron Microscopy Ltd, UK) operated at 5 kV accelerating voltage and 5 mm working distance.

### Cell survival

Number of cells attached to the implant were counted using a NucleoCounter at 72 h of stimulation. Briefly, each implant was removed from the PLA chamber and placed into a 2 mL Eppendorf tube. Lysis buffer (200 µL; Reagent A100, ChemoMetec A/S) was added and the tube was vortexed for 30 s to detach cells. Next, stabilisation buffer (200 µL; Reagent B, ChemoMetec A/S) was added and the tube was vortexed again for 30 s. The solution (detached cells and both buffer solutions) was taken up in a NucleoCounter cassette (NucleoCassette™, 941-0002) for counting.

### Collagen production

The amount of soluble collagen present in the cell culture medium at 72 h was measured using a collagen detection kit (Sircol Soluble Collagen Assay, Biocolor). The medium for every replicate in each experimental group was collected and diluted to 11.5 mL in consideration of uneven evaporation. Samples were prepared according to the manufacturer’s protocol and absorbance measurements were performed at 555 nm using a microplate reader (FLUOstar Omega, BMG LABTECH). OD_555nm_ values were transformed to μg collagen by the standard curve function, y = 5.1528 × x − 0.7766, R^2^ = 0.9665. Three technical replicates per sample were measured and each sample is presented as the mean value of the technical replicates.

### pH measurement

The pH of the cell culture medium was measured at 72 h using a pH meter (Beckman, USA). For each sample, the mean of two technical replicates was considered.

### Statistical analysis

One-way analysis of variance (ANOVA) with post hoc Tukey’s Honestly Significant Difference (HSD) test was used for statistical analysis, where *p* values < 0.05 were considered statistically significant. Mean values ± standard deviations are presented.
